# An unexpected response to adenosine

**DOI:** 10.1007/s12471-014-0631-z

**Published:** 2014-12-05

**Authors:** J. M. ter Maaten, R.G. Tieleman

**Affiliations:** Department of Cardiology, Martini Hospital Groningen, van Swietenplein 1, 9728 NT Groningen, the Netherlands

A 65 year-old lady was seen in our emergency department with palpitations, which had been present for several hours. She had never had palpitations before. Her medical history, as well as her family history, revealed no abnormalities. Physical examination showed haemodynamically stable values with an accelerated heart rate. The ECG is shown is Fig. 1. This ECG showed a narrow complex tachycardia with a rate of 133 beats/min with an intermediate electrical axis (Fig. 1). In lead V1 each QRS complex is preceded by a P wave with positive configuration. In the same lead there seems to be a notch in the R wave and possibly also in the T wave. These notches and P waves appear to display a regular pattern on closer examination. To differentiate between an atrial flutter with 2:1 block, an atrial tachycardia or an AV(N)RT, 6 mg of adenosine was administered. Paradoxically to what was expected, the rate of the tachycardia doubled to 270 beats/min, as shown in Fig. 2. What would be your diagnosis and what is the mechanism of the adenosine effect?

**Fig. 1:**
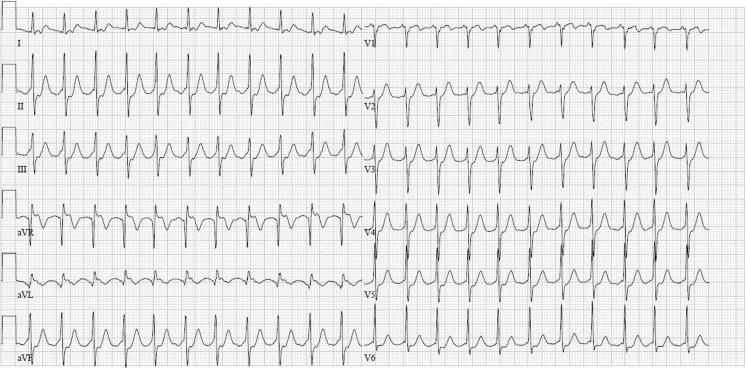
ECG at admission

**Fig. 2:**
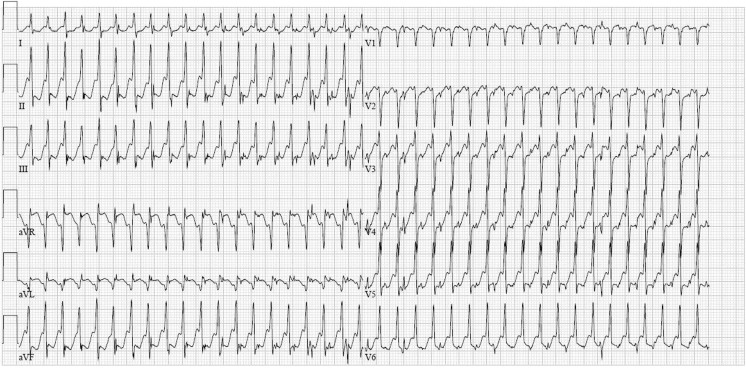
ECG after administration of adenosine

## Answer

You will find the answer elsewhere in this issue.

